# Psychosis and self-harm in prison: a population-based case–control study

**DOI:** 10.1136/bmjopen-2024-095464

**Published:** 2025-09-28

**Authors:** Nabila Z Chowdhury, Ye In (Jane) Hwang, Erin Spike, Azar Kariminia, Kimberlie Dean, Armita Adily, Andrew Ellis, David M Greenberg, Luke Grant, Stephen Allnutt, Tony Butler

**Affiliations:** 1 School of Population Health, University of New South Wales, Sydney, New South Wales, Australia; 2 The Kirby Institute, University of New South Wales, Sydney, New South Wales, Australia; 3 University of New South Wales, Sydney, New South Wales, Australia; 4 Justice Health and Forensic Mental Health Network, Matraville, New South Wales, Australia; 5 Corrective Services New South Wales, Sydney, New South Wales, Australia

**Keywords:** Schizophrenia & psychotic disorders, Suicide & self-harm, Forensic psychiatry, MENTAL HEALTH

## Abstract

**Abstract:**

**Background:**

Self-harm and suicide are common among prison inmates, but less is known about these phenomena in those with psychosis.

**Objectives:**

The aim of this study was to examine self-harm behaviour in New South Wales (NSW) prisons in Australia among inmates diagnosed with psychosis. This study also examined self-harm-related alerts applied by Corrective Services to assist staff with the management of the security and well-being of inmates.

**Design and setting:**

A retrospective case-control data-linkage study was conducted using administrative data collections in NSW, Australia.

**Participants:**

The study included all individuals diagnosed with psychosis and incarcerated between 2001 and 2020 in NSW as cases and an age and sex matched control group with no such diagnosis with a record of incarceration in the same time period.

**Primary and secondary outcome measures:**

The primary outcome measure was self-harm among the cases and controls. The secondary outcome measure was the application of alerts by Corrective Services in relation to self-harm incidents.

**Results:**

Multivariate regression analysis was used to examine predictors of self-harm in prison. Prisoners with psychosis (n=14 900) were more likely to self-harm than controls (n=2713), with 15.0% versus 3.6% engaging in self-harm (highest odds of self-harm observed in those with schizophrenia and related psychoses, aOR=4.84, 95% CI: 3.93 to 5.98). Those of Aboriginal heritage had an increased risk of self-harm (aOR=1.58, 95% CI: 1.43 to 1.75). Factors associated with a lower risk of self-harm were male sex and older age (≥25 years) at the time of their first incarceration. 35.6% of those released from prison with a prior psychosis diagnosis had at least one alert applied during incarceration compared with 10.1% of prisoners without a diagnosis of psychosis. Overall, 35 individuals with psychosis and 1 individual from the control group died while in prison between 2001 and 2020. 17 prison suicides were recorded from the study population; all occurred in the psychosis group.

**Conclusions:**

Given the heightened risk of self-harm in those with histories of psychosis, consideration should be given to sharing mental health information between agencies to improve the care and management of this group during incarceration. Prison alerts may be a useful tool to help staff manage inmates’ well-being if used appropriately.

**STRENGTHS AND LIMITATIONS OF THIS STUDY**

- This study used population-level, routinely collected data linked across the health and justice sectors.
- The use of objective administrative records reduced recall bias and allowed for large-scale, long-term follow-up.
- Data linkage allowed the identification of self-harm incidents in prison and mental health diagnoses across multiple sectors.
- Data on other psychiatric or medical comorbidities were unavailable, limiting adjustment for these factors.
- Alerts and self-harm incidents recorded by prison staff may be prone to human error, bias and a reluctance on the part of prisoners to report self-harm that likely underestimates the true extent of self-harm in prison.

## Background

 Self-harm is common among prisoners and is often linked to mental health difficulties and the psychological impact of incarceration. According to a systematic review and meta-analysis of 35 independent studies, 3.8% of 663 735 prisoners had a record of self-harm during incarceration, with a current psychiatric illness increasing the risk nearly eightfold.^1^

A study of detainees in New York City jails from January 2010 to January 2013 found self-harm to be associated with solitary confinement, serious mental illness, being aged ≤18 years and being Latino or White.^2^ Studies also indicate that prison suicide rates are higher than the rates in the general population. An Australian study reported 92 suicides during 1995–2005 in New South Wales (NSW) prisons, a rate 10 times higher than in the general population.^3^ According to a systematic review and meta-analysis of 77 studies, current psychiatric diagnosis (OR 6·4, 3·6–11·1) predicted suicide in prison.^4^ Two national prisoner surveys from England and Wales found that psychosis, neurosis and personality disorders were linked to suicidal thoughts and attempts, with younger age, single relationship status, being white, school dropout and poor social support associated with increased suicidal thoughts.^5^

Women prisoners appear to be at an increased risk of self-harm compared with men. A study from England and Wales found that self-harm rates in female prisoners were over 10 times higher than in men, with repeated self-harm being common in women under 20 years.^6^ A cross-sectional study of 996 NSW (Australia) prison inmates found that women and Aboriginal inmates were more likely to attempt suicide, with one-third reporting lifetime suicidal ideation.^7^

Prison screening and prevention programmes are key to preventing self-harm and suicide in correctional facilities. A review of 24 studies (1980–2022) found that suicide prevention programmes in correctional facilities significantly reduced suicide deaths (RR=0.35, 95% CI: 0.23 to 0.55), self-harm (Hedges’ g=−0.54) and suicidal ideation (Hedges’ g=−0.39), indicating their overall effectiveness despite variation in study designs and quality.^8^ An Australian study found that self-harm history and recent self-harm ideation were associated with self-harm in the following 12 months.^9^ In NSW, the Offender Information Management System (OIMS) is used by Corrective Services NSW (CSNSW) to manage offenders and includes a ‘prison alerts’ flag to notify staff and authorities about an offender’s status, behaviour or needs, including self-harm risk.^10^ However, little is known about the utility of the prison alert system used in NSW prisons in the prevention of self-harm and suicide.

## Objective

In this study, we examined risk factors associated with self-harm in prison in those diagnosed with psychosis and their control group in NSW, Australia. Self-harm refers to any intentional injury inflicted on oneself. We examined the use of the alert notification system applied during incarceration for self-harm and concerns for mental health for the study population. Suicides during incarceration are also reported.

## Methods

### Study design

This is a retrospective case-control study involving a population-level data linkage of several administrative data collections in NSW.

### Study population

#### Cases

143 506 individuals in NSW, Australia, were identified as having a diagnosis of psychosis on admission to hospital between July 2001 and June 2020, or an emergency department (ED) presentation between January 2005 and June 2020. Of these, 14 900 individuals had a custodial episode in a NSW prison between July 2001 and June 2020 and were considered cases in this study. The datasets included in case selection were the NSW Admitted Patient Data Collection (APDC), the NSW Emergency Department Data Collection (EDDC) and the NSW Offender Integrated Management System (OIMS) data collection.

#### Controls

Two age-matched and sex-matched individuals without any record of a psychosis diagnosis were randomly selected for each individual diagnosed with psychosis from the Centre for Health Record Linkage (CHeReL’s) control sampling frame using the random number generated by SAS 9.4 .^11^ Controls were matched to cases on year of birth and sex, and where the control’s death date was either unknown (NULL), or greater than the case index date. The datasets included in the creation of this group without a psychosis diagnosis were the NSW APDC, NSW EDDC, NSW Registry of Births, Deaths and Marriages (RBDM), NSW Mental Health Ambulatory Data Collection, NSW Perinatal Data Collection, NSW Cancer Registry, NSW Pap Test Registry and NSW Notifiable Conditions Information Management System. CHeReL provided an additional 12 565 unlinked individuals’ data, resulting in a total of 270 848 individuals without any diagnosis of psychosis. Of these, 2713 individuals had a custodial episode in NSW prisons during the same time period and were considered as controls in the current study, which excludes the unlinked controls, including only incarcerated individuals.

### Primary outcome

The primary outcome was at least one self-harm incident in prison as recorded in the NSW OIMS.

### Secondary outcome

The secondary outcome measures of this study were the application of alerts by Corrective Services in relation to self-harm incidents and suicides in prison in the study population.

### Data sources

Several de-identified whole population administrative data collections were accessed by the study. The NSW Ministry of Health’s APDC and EDDC were used to select the study population. The APDC includes records for all hospital separations from NSW public and private hospitals, including day procedure centres. The EDDC provides information about presentations to public hospital EDs. We extracted clinical data, including psychosis diagnosis and treatment episodes dates, as well as sociodemographic data such as gender, age, indigenous status, marital status and socio-economic indexes of areas (SEIFA) of living at the time of the most recent psychosis diagnosis for cases, and the most recent record for controls from both the data collections between July 2001 to June 2020.

The NSW OIMS holds personal, current, previous offence and incarceration data for all persons 18 years or older who have spent time in prison. We used this data collection to retrieve information on incarceration episodes for the study period. OIMS also records key management information such as prisoner alerts and self-harm incidents reported and verified by registered prison officers. We extracted data on the type of alert, alert date and date of self-harm from OIMS for the same time period.

Data on overall deaths and specifically on suicide were extracted from the NSW RBDM and the Cause of Death Unit Record File (COD URF), respectively, which is held by the Australian Bureau of Statistics. Suicide was coded using the International Classification of Diseases version 10 (ICD-10). Both the data collections pertained to the period July 2001 to June 2019.

Table 1 shows all the data sources used in this study and the dates of data collection:

**Table 1:** Data collections used in the study

Dataset source	Dates used for the study
NSW Admitted Patient Data Collection (APDC)	July 2001–June 2020
Emergency Department Data Collection (EDDC)	January 2005–June 2020
NSW Offender Information Management System (OIMS)	July 2001–June 2020
NSW Registry of Births, Deaths and Marriages (RBDM)	July 2001–June 2019
Cause of Death Unit Record File (COD URF)	July 2001–June 2019

NSW, New South Wales.

### Covariates

We ascertained gender using the most commonly recorded value within and across the APDC, EDDC, OIMS, RBDM and COD-URF datasets. Aboriginal status was determined based on any record indicating Aboriginality (Aboriginal or Torres Strait Islander status) in the service contact data, using the most frequent value within and across APDC, EDDC, OIMS, RBDM and COD-URF. Age at first prison episode was calculated using the date of birth and the start date of the first prison episode based on APDC, EDDC and OIMS data and grouped as follows: <25, 25–34, 35–44 and ≥45 years. Marital status (married or single) was sourced from the APDC, with ‘married’ including de facto relationships, and ‘single’ including those widowed, divorced or permanently separated. SEIFA was derived from both the APDC and EDDC. Socio-economic status was assigned based on the index of relative socio-economic disadvantage, which ranks areas according to relative disadvantage using statistical local areas.^12^ The lowest rank indicates the most disadvantaged areas, and the highest rank the most advantaged. We categorised areas into disadvantaged (lower 5 deciles 1–5) and advantaged (upper 5 deciles 6–10). We dichotomised SEIFA at the median (50–50 split) to create balanced groups for clearer comparison and improved statistical stability between relatively lower and higher socio-economic disadvantage.

### Data linkage

Data linkage was performed by the NSW CHeReL to ensure the anonymity of the data provided to the researchers. Identifying information such as name, address, date of birth and gender for each dataset is included in the Master Linkage Key, which was constructed by CHeReL using probabilistic record linkage methods and ChoiceMaker software.^13^ No health/content data are used in the linkage process. CHeReL created a Project Person Number (PPN) for each person identified in the linkage and assigned the PPNs to every data set used for the study. The CHeReL returned the PPN and the encrypted record number from the source datasets to data custodians who independently supplied the datasets to the researchers.

### Definitions

#### Psychosis

Psychosis was defined as having had a diagnosis of psychosis in a whole episode of care by a clinician on admission to a hospital or presentation to an ED recorded during the study period. Diagnoses from the APDC were coded according to the ICD 10th Revision (ICD-10) and diagnoses from the EDDC were coded according to the ICD 9th Revision (ICD-9) or 10th Revision (ICD-10) or the systematised nomenclature of medicine–clinical terms.^14^ We divided psychosis into three categories:

- Schizophrenia and related psychoses: ICD-10 diagnostic codes F06.0, F06.2, F20.0, F20.1, F20.2, F20.3, F20.4, F20.5, F20.6, F20.8, F20.9, F22.0, F22.8, F22.9, F23.0, F23.1, F23.2, F23.3, F23.8, F23.9, F24, F25.0, F25.1, F25.2, F25.8, F25.9, F28, F29 and F53.1; ICD-9 diagnostic codes 293.81, 293.82, 295.0, 295.1, 295.2, 295.3, 295.4, 295.6, 295.7, 295.8, 295.9, 297.0, 297.1, 297.2, 297.3, 297.8, 297.9, 298.3, 298.4, 298.8 and 298.9.
- Affective psychoses: ICD-10 diagnostic codes F30.2, F31.2, F31.5, F32.3 and F33.3; ICD-9 diagnostic codes 296.04, 296.14, 296.24, 296.34, 296.44, 296.54, 296.64 and 298.0.
- Substance-related psychoses: ICD-10 diagnostic codes F10.5, F11.5, F12.5, F13.5, F14.5, F15.5, F16.5, F17.5, F18.5 and F19.5; ICD-9 diagnostic codes 291.3, 291.5, 292.1, 292.11 and 292.12

We used a hierarchical approach to categorise psychosis: those with a diagnosis of schizophrenia and related psychoses were coded as ‘schizophrenia and related psychoses’, diagnoses of affective psychoses without schizophrenia and related psychoses were coded as ‘affective psychoses’ and substance-related psychoses without the other two groups were coded as ‘substance-related psychoses’.

#### Prison alerts

Alerts are created in the OIMS when a CSNSW custodial officer or other staff member finds it necessary to do so due to a particular incident or because of an inmate’s behaviour or physical or mental health at any time point during the prison episode. The purpose of the alert is to help staff manage the security and well-being of inmates.^10^ According to CSNSW, ‘*when an inmate is deemed to be at risk of self-harm, an alert must be entered into the OIMS by the officer in charge once they have been informed by the reporting officer. This alert will reflect a ‘pending status’ until it is reviewed by the Risk Intervention Team (RIT). It then becomes the responsibility of the coordinator of the RIT to verify the alert*.^10^

This study focused on mental health-related alerts and those concerning self-harm.

### Statistical analysis

Frequencies, percentages and χ^2^ tests of association were used to describe categorical variables. Continuous variables were described using median (IQR) due to skewed distributions observed in histograms and Q-Q plots. Multivariable logistic regression was used to examine predictors of self-harm incidents in prison. Statistically significant covariates (p<0.05) were included in the model using a forward stepwise approach. Both combined and gender-stratified analyses were conducted for descriptive and logistic regression analysis. Prison alerts, self-harm and suicide were also reported only in study cohorts with completed prison episodes. Missing data were assumed to be missing at random, as missingness was associated with observed sociodemographic variables rather than unobserved outcomes. We created a separate category for missing or unknown values. All analyses were completed using SAS V.9·4 and STATA V.16.

Strengthening the Reporting of Observational Studies in Epidemiology, case-control reporting guidelines were used throughout the manuscript.^15^

### Patient and public involvement in the study

Patients and the public were not involved in the design, conduct, reporting or dissemination of this study. The study used routinely collected, de-identified administrative data collections, and there was no direct participant interaction.

### Ethics

Approvals for the project were received from NSW Population and Health Services Research Ethics Committee (HREC/15/CIPHS/17), Justice Health and Forensic Mental Health Network (G324/14), Corrective Services NSW (D15/138715) and the NSW Aboriginal Health and Medical Research Council (1089/15).

## Findings

### Characteristics of the study population

Overall 14 900 cases with psychosis and 2713 matched controls were incarcerated at least once between July 2001 and June 2020 (table 2). A high proportion (84.4%) of the study population was male. The distribution of cases and controls in terms of age at first imprisonment followed a similar trend. First imprisonment for the study cohort was most common in ages under 25 years (34.4% cases vs 30.1% controls) and least common among individuals 45 years or older (8.2% cases vs 11.0% controls). 24.5% of cases were of Aboriginal heritage compared with 20.2% of controls. A higher proportion of controls reported married or de facto relationship status (22.0% vs 11.5%), and being from a disadvantaged area was less common in controls compared with cases (62.8% vs 69.5%).

**Table 2:** Demographic characteristics of cases and controls, including by sex—New South Wales (July 2001–June 2020)

Characteristics	Total* (n=17 613)		Men (n=14 869; 84.4%)			Women (n=2744; 15.6%)		
	Cases (n=14 900; 840.6%)	Controls (n=2713; 15.4%)	Cases (n=12 367; 830.2%)	Controls (n=2502; 16.8%)	P value	Cases (n=2533; 92.3%)	Controls (n=211; 7.7%)	P value
Age at first prison episode	n (%)	n (%)	n (%)	n (%)	<0.001	n (%)	n (%)	<0.001
<25 years	5121 (34.4)	817 (30.1)	4355 (35.2)	761 (30.4)		766 (30.2)	56 (26.5)	
25–34 years	5593 (37.5)	966 (35.6)	4579 (37.0)	909 (36.3)		1014 (40.0)	57 (27.0)	
35–44 years	2970 (19.9)	633 (23.3)	2426 (19.6)	560 (22.4)		544 (21.5)	73 (34.6)	
45+ years	1216 (8.2)	297 (11.0)	1007 (8.1)	272 (10.9)		209 (8.3)	25 (11.9)	
Indigenous status					0.016			0.017
Yes	3647 (24.5)	548 (20.2)	2740 (22.2)	491 (19.6)		907 (35.8)	57 (27.0)	
No	11 235 (75.4)	2162 (79.7)	9612 (77.7)	2009 (80.3)		1623 (64.1)	153 (72.5)	
Missing/unknown	18 (0.1)	3 (0.1)	15 (0.1)	2 (0.1)		3 (0.1)	1 (0.5)	
Marital status					<0.001			0.027
Married	1717 (11.5)	596 (22.0)	1360 (11.0)	555 (22.2)		357 (14.0)	41 (19.4)	
Single	10 802 (72.5)	1663 (61.3)	9035 (73.1)	1516 (60.6)		1767 (69.8)	147 (69.7)	
Missing/unknown	2381 (16.0)	454 (16.7)	1972 (15.9)	431 (17.2)		409 (16.2)	23 (10.9)	
SEIFA					<0.001			0.021
Advantaged	4621 (31.0)	751 (27.7)	3824 (30.9)	692 (27.7)		797 (31.4)	59 (28.0)	
Disadvantaged	9359 (62.8)	1886 (69.5)	7751 (62.7)	1737 (69.4)		1608 (63.5)	149 (70.6)	
Missing/unknown	920 (6.2)	76 (2.8)	79	73 (2.9)		128 (5.1)	3 (1.4)	

* All significant differences between total cases and total controls (p<0.001, χ2 test).

SEIFA, socio-economic indexes of areas.

The distribution of characteristics was similar between men and women. However, a greater proportion of women in the control group were aged between 35 and 44 years compared to cases (34.6% vs 22.4%). Among both cases and controls, a greater proportion of women were Aboriginal compared with men (35.8% vs 22.2% in cases; 27.0% vs 19.6% in controls), and among cases, a higher proportion of women were married/in a de facto relationship compared with men (14.0% vs 11.0%).

### Self-harm in prison

Overall, 15.0% (n=2230) of those with psychosis had a record of self-harm while in prison compared with 3.6% (n=98) of controls across the years 2001–2020. This included self-harm episodes across all prison episodes, regardless of the timing of the psychosis diagnosis.

Table 3 presents the factors associated with an increased risk of self-harm, showing adjusted aORs and 95% CIs. All estimates are mutually adjusted for the variables listed in the table. Overall, there was an increased risk of self-harm for those with psychosis compared with those without psychosis, with the highest odds observed in those with schizophrenia and related psychoses (aOR=4.84, 95% CI: 3.93 to 5.98, p<0.001) (table 3). The risk was also higher among those identifying as Aboriginal (aOR=1.58, 95% CI: 1.43 to 1.75, p<0.001). A lower risk of self-harm was observed in males compared with females (aOR=0.72, 95% CI: 0.64 to 0.81, p<0.001), older individuals compared with those aged <25 years during first imprisonment (the lowest aOR occurred in those with age ≥45 years; aOR=0.25, 95% CI: 0.20 to 0.32, p<0.001). We found no significant association of self-harm with marital status and SEIFA.

In the gender-stratified analysis, similar patterns were observed both in men and women. In women, however, affective psychosis (aOR=2.62, 95% CI: 1.00 to 6.91, p=0.051) and Aboriginal heritage (aOR=1.23, 95% CI: 1.00 to 1.51, p=0.055) were associated with increased odds of self-harm, although these associations did not reach conventional statistical significance. Women from disadvantaged SEIFA showed a significant association with self-harm (aOR=1.29, 95% CI: 1.02 to 1.63, p=0.031).

**Table 3:** Adjusted aOR for self-harm in prison—July 2001–June 2020

	Overall (n=17 613)			Men (n=14 869; 84.4%)		Women (n=2744; 15.6%)	
	Self-harm n (%)	aOR (95% CI)	P value	aOR (95% CI)	P value	aOR (95% CI)	P value
Psychosis							
No	98 (3.6)	1		1		1	
Schizophrenia and related psychoses	1921 (16.3)	4.84 (3.93 to 5.98)	<0.001	4.77 (3.83 to 5.95)	<0.001	5.71 (2.83 to 11.10)	<0.001
Affective psychosis	52 (11.5)	3.72 (2.60 to 5.32)	<0.001	4.17 (2.83 to 6.15)	<0.001	2.62 (1.00 to 6.91)	0.051
Substance-related psychosis	257 (9.6)	2.51 (1.97 to 3.19)	<0.001	2.48 (1.91 to 3.21)	<0.001	2.82 (1.36 to 5.86)	0.006
Gender							
Female	487 (17.7)	1		–		–	
Male	1841 (12.4)	0.72 (0.64 to 0.81)	<0.001	–	–	–	–
Indigenous status							
No	1514 (11.3)	1		1		1	
Yes	813 (19.4)	1.58 (1.43 to 1.75)	<0.001	1.70 (1.52 to 1.90)	<0.001	1.23 (1.00 to 1.51)	0.055
Missing/unknown	1 (4.8)	0.39 (0.05 to 2.95)	0.359	1	–	1.92 (0.16 to 23.27)	0.609
Age groups at first prison episode							
<25 years	1169 (19.7)	1		1		1	
25–34 years	787 (12.0)	0.56 (0.51 to 0.63)	<0.001	0.60 (0.54 to 0.67)	<0.001	0.44 (0.35 to 0.55)	<0.001
35–44 years	290 (8.0)	0.37 (0.32 to 0.43)	<0.001	0.38 (0.32 to 0.44)	<0.001	0.34 (0.25 to 0.46)	<0.001
45+ years	82 (5.4)	0.25 (0.20 to 0.32)	<0.001	0.28 (0.22 to 0.37)	<0.001	0.14 (0.07 to 0.25)	<0.001
Marital status							
Married	290 (12.5)	1		1		1	
Single	1728 (13.9)	0.89 (0.77 to 1.01)	0.072	0.86 (0.74 to 1.01)	0.069	0.95 (0.70 to 1.27)	0.707
Missing/unknown	310 (10.9)	0.73 (0.62 to 0.88)	0.001	0.69 (0.56 to 0.84)	<0.001	0.94 (0.64 to 1.36)	0.732
SEIFA							
Advantaged	660 (12.3)	1		1		1	
Disadvantaged	1507 (13.4)	1.00 (0.91 to 1.11)	0.949	0.94 (0.84 to 1.05)	0.286	1.29 (1.02 to 1.63)	0.031
Missing/unknown	161 (16.2)	1.27 (1.05 to 1.54)	0.015	1.25 (1.01 to 1.54)	0.037	1.31 (0.80 to 2.12)	0.280

SEIFA, socio-economic indexes of areas.

### Alerts in completed prison episodes (2001–2020) in prisoners with a prior diagnosis of psychosis

**Figure 1:**
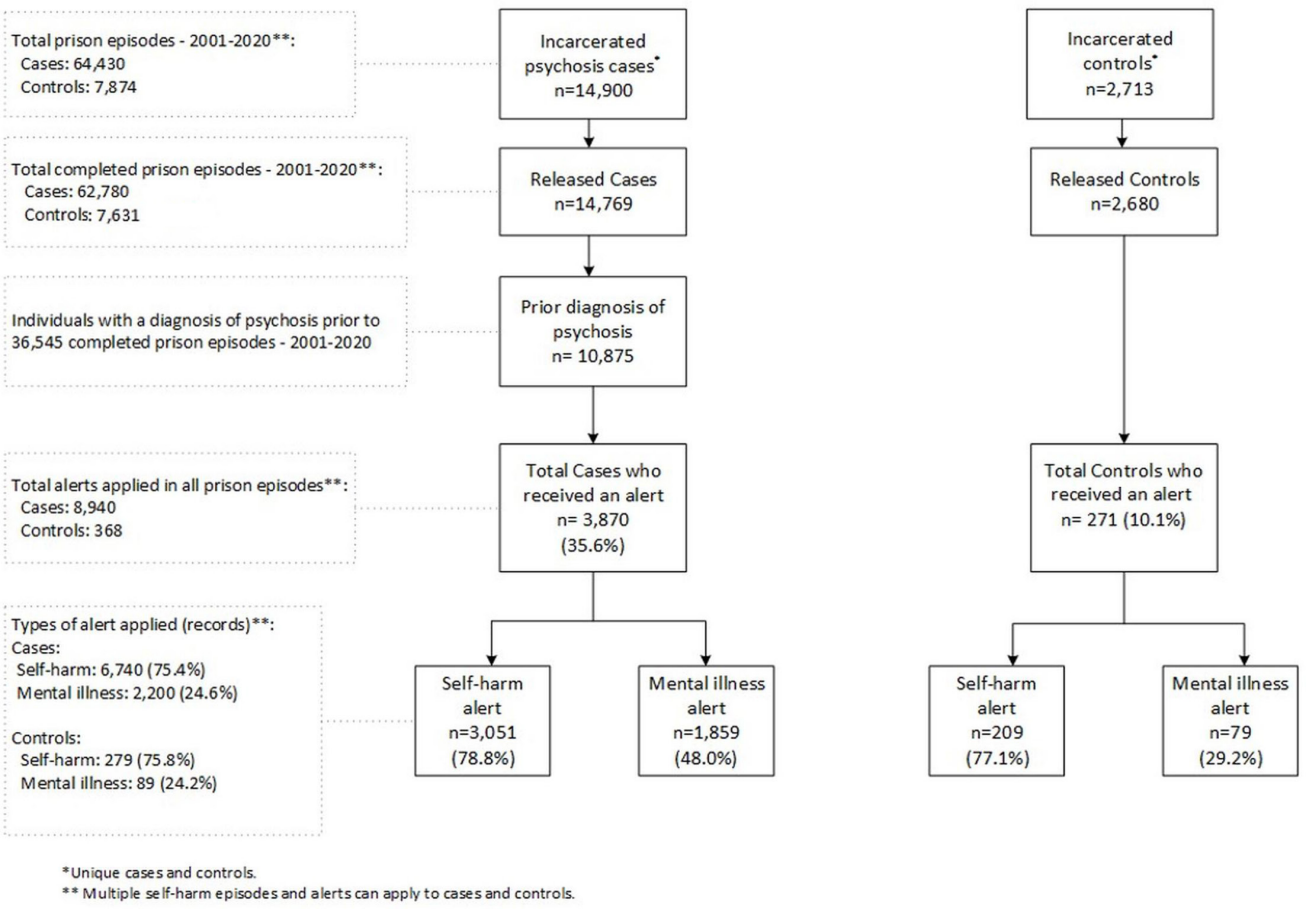
Alerts in completed prison episodes for individuals with a prior diagnosis of psychosis and controls (2001–2020).

Among the 14 900 cases and 2713 controls who had an incarceration episode between 2001 and 2020, 14 769 (99.1%) cases and 2680 (98.8%) controls had at least one completed prison episode and had been released from prison during this period (figure 1). We selected cases where the diagnosis of psychosis occurred prior to entering prison (n=10 875). During a total of 36 545 prison episodes for those with a prior diagnosis of psychosis, 8940 alerts were applied to more than one-third of cases (n=3870; 35.6%) compared with 10.1% (n=271) of controls who had alerts during 7631 prison episodes (p<0.001) (figure 1).

Among 3870 cases with at least one alert applied, 6740 alerts (75.4%) were applied for self-harm to 3051 cases (ie, 78.8% of cases), and 2200 mental illness alerts (24.6%) were applied to 1859 cases (ie, 48.0% of cases). A similar distribution of alert types was applied to controls: 75.8% for self-harm (applied to 77.1% controls) and 24.2% for mental illness (applied to 29.2% controls). As might be expected, a higher proportion of psychosis cases had an alert for mental illness compared with controls (48.0% vs 29.2%, p<0.001).

### Self-harm and suicide during the most recent incarceration

**Figure 2:**
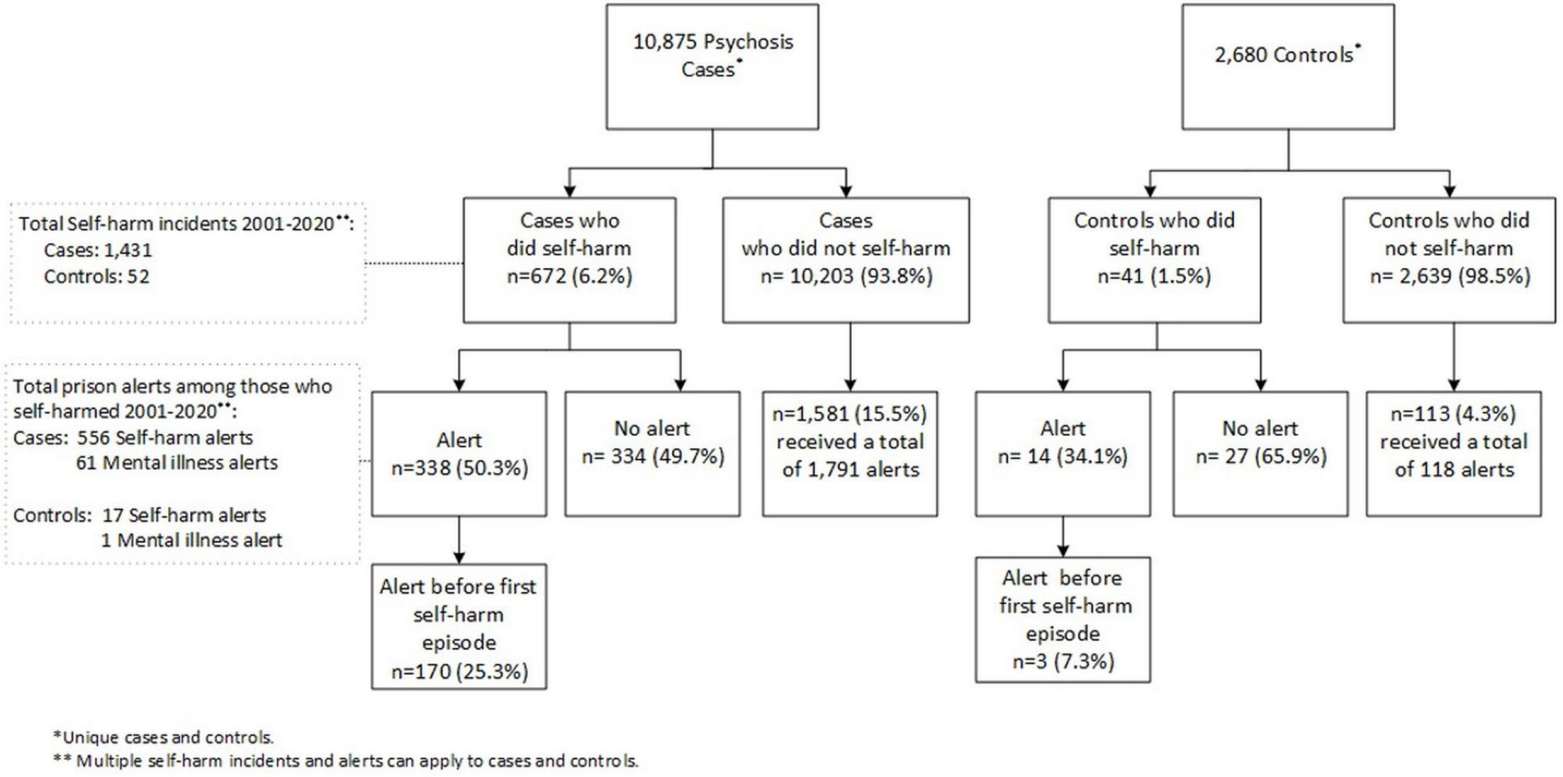
Self-harm in relation to prison alerts (for self-harm and mental illness) during the last incarceration (2001–2020).

In the most recent completed prison episodes, a total of 1431 self-harm incidents were recorded for 6.2% of cases and 1.5% of controls (p<0.001) (figure 2). The median time to the first self-harm incident from entering prison was 27 days among cases (IQR: 6–84 days) and 19 days among controls (IQR: 2–75 days). Among cases who did self-harm, 338 individuals (50.3%) had a total of 556 self-harm alerts and 61 mental illness alerts applied, whereas 14 controls (34.1%) had 17 self-harm alerts and 1 mental illness alert applied.

Overall, cases and controls who had a record of self-harm received more alerts compared with those with no such record (50.3% vs 15.5% in cases and 34.1% vs 4.3% in controls, p<0.001) (figure 2). Of the cases who did self-harm, 25.3% had an alert (n=170) before their first self-harm incident on that prison episode compared to 7.3% of controls (n=3) (p<0.001).

Overall, from this study cohort, a total of 1209 cases and 132 controls died up until June 2019. Among these, 35 individuals with psychosis (0.23% of cases) died while in prison compared with one control (0.04%), indicating almost a 6-fold higher rate of in-prison deaths among cases compared with controls, although this difference was not statistically significant (p=0.25). A total of 17 suicides were reported in prison among the study population, all occurring in the psychosis group. Out of 17 suicides, six individuals had a self-harm alert, and one individual had a mental illness alert in the same prison episode when the suicide occurred.

## Discussion

Overall, we found that individuals with psychosis were significantly more likely to self-harm during incarceration compared with those with no such diagnosis (15% vs 3.6%), with women having a higher risk than men. The highest odds of self-harm were observed in those with schizophrenia and related psychoses compared to those with other forms of psychosis. These results provide compelling evidence that individuals with psychosis are extremely vulnerable during incarceration and that chronic forms of psychosis carry the greatest risk.

The prevalence of self-harm in the control group with no history of psychosis was 3.6%, which is similar to the pooled result (3.8%) of a systematic review of 35 independent studies comprising a total of 663 735 prisoners.^1^ A cross-sectional descriptive study in the US, which included all inmates between 2005 and 2010, reported that those with a mental health disorder (anxiety, bipolar, depression and psychotic disorders) were significantly more likely (9.2 times greater odds) to attempt suicide inside prison compared with those who had no such disorders.^16^ Although we did not report the odds of suicide or attempted suicide in this study, all observed suicides (n=17) in prison within our study population occurred in the psychosis group, showing the extreme vulnerability of this group.

Aboriginal people were at greater risk of self-harm in prison. Conversely, being male and aged 25 or above decreased the risk of self-harm. Previous research has also identified that self-harm is more prevalent in women and younger inmates compared to men and older inmates, respectively.^6^

Providing adequate mental health support during incarceration and using mental health alerts to inform caregivers and management in prison may play a crucial role in preventing self-harm and suicide. However, our findings show that alerts are inconsistently used in NSW prisons, with only 35.6% of released inmates (2001–2020) with a prior psychosis diagnosis having an alert, compared to 10.1% of controls. More than two-thirds of all alerts given to individuals with psychosis were for self-harm. The proportion of alerts can serve as a proxy for the effectiveness of in-prison screening procedures, and our findings suggest this metric falls short of satisfactory for individuals with histories of psychosis who are incarcerated. Previous research has suggested scope for enhancement of the prison screening system.^17 18^ Managing inmates with serious health problems is complex for custodial and health services. Custodial and correctional staff and systems working alongside health professionals/systems is a necessity in this regard. Implementing specialised training for prison staff to recognise early signs of severe mental illness, ensuring clinicians follow up after routine mental health screenings and developing crisis intervention teams within prisons could enhance the screening system.

Our study suggests that the initial days of imprisonment represent a time of heightened vulnerability for new inmates, with enhanced monitoring for all inmates essential during this period. Our results show that nearly 50% of individuals with psychosis who had a record of self-harm during their most recent prison episode lacked any mental health or self-harm alert in the same episode, indicating an inefficient alert system for self-harm prevention. This highlights the importance of sensitive data sharing between healthcare services and those managing individuals with mental illness in prison. Access to psychiatric history could potentially save lives by improving care and management. Precedents exist for health information sharing in the area of infectious diseases, such as occurs for COVID-19 and tuberculosis, where information is shared with the correctional system in the interest of public health within prisons. Such information sharing for mental health conditions would require checks and balances to ensure its appropriate use and to prevent those flagged by the system from experiencing any negative repercussions.

A total of 36 individuals in our study population died while in prison between July 2001 and June 2019, with 35 belonging to the psychosis group. All suicides (n=17) occurred in the psychosis group, with approximately one-third flagged on the alerts system during the same prison episode prior to death. This suggests that prison alerts may be important for identifying future suicide risk, indicating that enhanced monitoring of at-risk individuals is necessary to reduce this risk. A systematic review on suicide prevention in psychiatric inpatients suggested that both prison staff and inmates debriefing after suicide, staff training programmes, ongoing screening, improved staff communication and community-based mental health services are crucial for preventing future suicides among inmates.^19^

Addressing self-harm among prisoners with mental illness is a complex and sensitive issue that requires a comprehensive and empathetic approach. Further research is needed on the effectiveness of measures taken by prisons in response to self-harm risk indicators and mental illness diagnoses. This should include examining the availability of mental health treatment and communication between prison inmates and staff. Prison alerts, coupled with suicide prevention measures and rehabilitation programmes tailored to the needs of mentally ill inmates, can help reduce the likelihood of reoffending, thereby improving the chances of successful reintegration into society. Fostering awareness and understanding of mental health issues, implementing effective identification and screening processes, and providing comprehensive training for prison staff create an environment that supports at-risk inmates. Previous research showed that the challenges in managing prisoners at risk of suicide can worsen due to communication gaps between departments, and without addressing prison staff perceptions and attitudes, efforts to enhance procedures may not yield significant improvements.^20^

## Limitations

This study did not include those diagnosed with psychosis exclusively in private clinics or by general practitioners, though most of those with psychosis are treated by public services at some point, so the impact of this on our findings is likely to be small. Our study only included records for NSW, so those diagnosed with psychosis or convicted of a crime in another state or overseas would not have been included. Alerts and self-harm incidents recorded by prison staff may be subject to reporting bias, underreporting and human error, which could affect the accuracy and completeness of the data. This study also lacked data on other psychiatric or medical comorbidities beyond psychosis, which limits our ability to account for their potential influence on the findings. This study was unable to distinguish between non-suicidal self-injury and actual suicide attempts with intent, which is another limitation of the available data.

## Conclusion and Implications

In conclusion, individuals with psychosis were more likely to self-harm in prison than those without psychosis. The study suggests that prison alerts could play a key role in recognising future self-harm risk, including suicide, among inmates with psychosis.

More focused attention and care in prison can lead to better management of the mental health needs of inmates with psychosis, which may help identify warning signs of self-harm earlier among them, which ultimately can reduce the likelihood of such incidents.
