# Supplementary material for: Psychosis and self-harm in prison: a population-based case–control study
Source: BMJ Open. 2025 Sep 28;15(9):e095464. doi: 10.1136/bmjopen-2024-095464 (PMC12481374; doi:10.1136/bmjopen-2024-095464)
Supplement: Supplementary file 1 [file bmjopen-2024-095464.reviewer_comments.pdf]

PEER REVIEW HISTORY

BMJ Open publishes all reviews undertaken for accepted manuscripts. Reviewers are asked to complete a checklist review form and are provided with free text boxes to elaborate on their assessment. These free text comments are reproduced below.

ARTICLE DETAILS

Title (Provisional)

Psychosis and self-harm in prison: A Population-based Case-Control study

Authors

Chowdhury, Nabila Z.; Hwang, Ye In (Jane); Spike, Erin; Kariminia, Azar; Dean, Kimberlie; Adily, Armita; Ellis, Andrew; Greenberg, David M; Grant, Luke; Allnutt, Stephen; Butler, Tony

---

VERSION 1 - REVIEW

**Reviewer**	**1**
**Name**	**Zai, Clement C**
**Affiliation**	**Campbell Family Mental Health Research Institute**
**Date**	**16-Dec-2024**
**COI**	**None**

This is an important study on risk factors of self-harm and suicide prisoners in NSW with focus on psychosis. The results were presented clearly and the discussions and limitations were appropriate. However, it may be improved by addressing the following:

- In the abstract, the "controls without psychosis" can be clarified as prisoner without psychosis. For the "35.6%" about alerts and 17 suicide deaths for prisoners with psychosis, the corresponding numbers for prisoners without psychosis can be given.

- In the introduction, it would be helpful to include the effect estimates from the meta-analysis of suicide prevention programs in correctional facilities.

- In the Methods, the matching with sex and age was done for the 143,506 cases? What was the age range for each match? Secondary outcomes should also be stated in the Methods. The rationale for dichotomizing SEIFA should be provided (with at least a reference). It is unclear if all tested predictors have been included in Tables 1 and 2; in addition, all predictors should be

included in the Methods. Gender-stratified analyses should have been mentioned in the analysis methods. Analysis methods for alerts need to be included.

- Sensitivity analyses on only participants with complete data should be carried out.

- In the Results, the effect estimates and p-values for comparisons made regarding alerts and the most recent incarcerations need to be included.

- In the Discussion, the authors should clarify the impact of diagnosis made in private clinics, general practice, vs public services on the findings and how this impact was "likely small". The authors should also mention the lack of information to differentiate between non-suicidal self-injuries and suicide attempts with intent as a limitation. The authors could also elaborate on the comorbidities (limitations) in terms of psychiatric / medical conditions and consideration of these conditions in cases and controls.

**Reviewer**	**2**
**Name**	**Kelson, Zoe**
**Affiliation**	**University of Exeter, Mathematics**
**Date**	**03-Jun-2025**
**COI**	**None**

The aim of this retrospective case-control data-linkage study was to examine self-harm behaviour in New South Wales (NSW) prisons in Australia among inmates diagnosed with psychosis from 2001 to 2020.

Reviewer comments:

"143,506 cases were identified as individuals in NSW, Australia, diagnosed with psychosis on admission to hospital between July 2001 and June 2020 or an emergency department between June 2005 and June 2020. For this study, we selected 4,900 individuals from cases s who had a custodial episode in NSW prisons during July 2001 and June 2020."

Can the authors please further comment on whether they consider the included cohort to be representative?

"Two age- and sex-matched controls without any record of psychosis diagnosis were randomly selected for each case from CHeReL's (Centre for Health Record Linkage) control sampling frame"

Can the authors please specify the matching algorithm or process they followed here?

"CHeReL also provided an additional 12,565 unlinked controls resulting in a total of 270,848 individuals as controls without any diagnosis of psychosis."

and

"For this study, we selected 2,713 controls who had a custodial episode in NSW prisons during the same time period. "

Can analyses please also be stratified by control type (i.e. matched and unlinked)?

"The primary outcome of this study was at least one self-harm incident in prison as recorded in the NSW Offender Integrated Management System (OIMS). We also examined prison alerts for self-harm incidents including suicide in prison."

Can the authors please confirm whether all incidents of self-harm and suicide have been captured in this data?

"Continuous variables were described using median and interquartile range (IQR)."

Can the authors please clarify if (and how) distributional assumptions were checked prior to the application of non-parametric statistical descriptors?

"Multivariate regression analysis was used to examine predictors of self-harm in prison." [Abstract]

and

"Multivariable logistic regression was used to examine predictors of self-harm incidents in prison. Statistically significant covariates ($p<0.05$) were included in the model using a forward stepwise approach."

Appropriate modelling methods have been applied by the authors.

Can the authors please specify (i.e. list) the covariates included in the model in the methods section?

"Unknown/missing values were combined with the most common category."

Can the authors please comment on whether missing/unknown values can be assumed to be missing at random?

Can the authors please conduct sensitivity analyses on the approach taken for handling missing data (e.g. undertaking a complete case analysis and performing multiple imputation)?

"STROBE case-control reporting guidelines were used throughout the manuscript"

Thanks for providing a copy of the STROBE checklist in the supplementary material.

"Overall, 14,900 cases with psychosis and 2,713 matched controls were incarcerated at least once between July 2001 and June 2020 (Table 1)."

and

"24.5% of cases were of Aboriginal heritage compared to 20.2% of controls. A higher proportion of controls reported having married or de facto relationship status (22.0% vs. 11.5%) and being from a disadvantaged area was less common in controls compared to cases (62.8% vs. 69.5%)...

a greater proportion of women in the control group were aged between 35-44 years compared to cases (34.6% vs. 22.4%). Among both cases and controls, a greater proportion of women were Aboriginal compared to men (35.8% vs. 22.2% in cases; 27.0% vs. 19.6% in controls); and among cases, a higher proportion of women were married/in a de facto relationship compared to men (14.0% vs. 11.0%)"

Can the authors please confirm whether all significantly different variables between groups have been adjusted for in the models?

"35 individuals with psychosis died in prison between 2000 and 2020; 17 from suicide" [Abstract]

and

"A total 17 suicides were reported inside prison among the study population and all of the suicides occurred in the psychosis group"

Can the fact that 'all of the suicides occurred in the psychosis group' please be clarified in the Abstract?

Can the number of individuals without psychosis who died in prison between 2000 and 2020 please also be reported (as proportions)?

Table 2: "adjusted by psychosis type, age, sex, and Aboriginal background "

Can the table heading please specify that Marital status and Socio-Economic Indices for Areas have also been adjusted for in the models?

"This study excluded those diagnosed with psychosis exclusively in private clinics or by general practitioners, though most are treated by public services at some point, so the impact is likely small. It only includes NSW records, so those diagnosed with psychosis or convicted of a crime interstate or overseas would have been missed. Alerts and self-harm incidents recorded by prison staff may be prone to human error, bias, and reporting reluctance can affect accuracy. The study also lacks data on ethnic groups, employment/education history, and other comorbidities."

A discussion on the study limitations has been provided.

VERSION 1 - AUTHOR RESPONSE

Reviewer 1: Dr. Clement C Zai, Campbell Family Mental Health Research Institute

Comments to the Author:
This is an important study on risk factors of self-harm and suicide prisoners in NSW with focus on psychosis. The results were presented clearly and the discussions and limitations were appropriate. However, it may be improved by addressing the following:

1. **In the abstract, the "controls without psychosis" can be clarified as prisoner without psychosis.**

Author's Response:
We thank the reviewer and have made changes in the abstract in the revised manuscript as follows:

> **Page 1:**
> "The study included all individuals diagnosed with psychosis and incarcerated between 2001 and 2020 in NSW as cases and their age and sex matched control group with no such diagnosis with record of incarceration at the same time period."

Also note that the parent cohort includes those with psychosis and age and sex matched individuals without a psychosis diagnosis regardless of incarceration status. We have made this clearer in the study population section in the revised manuscript as follows:

> **Page 4:**
> "**Study population**
> *Cases:* 143,506 individuals in NSW, Australia were identified as having a diagnosis of psychosis on admission to hospital between July 2001 and June 2020 or an emergency department contact between January 2005 and June 2020. Of these, 14,900 individuals had a custodial episode in a NSW prison between July 2001 and June 2020 and considered as cases in this study. The datasets included in case selection were the NSW Admitted Patient Data Collection (APDC) and NSW Emergency Department Data Collection (EDDC).
>
> *Controls:* Two age- and sex- matched individuals without any record of a psychosis diagnosis were randomly selected for each individual diagnosed with psychosis from the Centre for Health Record Linkage (CHeReL's) control sampling frame using the random number generated by SAS [11]. Controls were matched to cases on year of birth and sex, and where the control's death date was either unknown (NULL), or greater than the

case index date. The datasets included in the creation of this group without psychosis diagnosis were the NSW APDC, NSW EDDC, NSW Registry of Births, Deaths and Marriages (RBDM), NSW Mental Health Ambulatory Data Collection, NSW Perinatal Data Collection, NSW Cancer Registry, NSW Pap Test Registry, and NSW Notifiable Conditions Information Management System. CHeReL provided an additional 12,565 unlinked individual's data resulting in a total of 270,848 individuals as without any diagnosis of psychosis. Of these, 2,713 individuals had a custodial episode in NSW prisons during the same time period and considered as controls in this current study."

2. **For the "35.6%" about alerts and 17 suicide deaths for prisoners with psychosis, the corresponding numbers for prisoners without psychosis can be given.**

Author's Response:
We agree and have revised the abstract in the revised manuscript as follows:

Page 2:
"35.6% of those released from prison with a prior diagnosis of psychosis had at least one alert applied during incarceration compared to 10.1% of prisoner without a diagnosis of psychosis. Overall, 35 individuals with psychosis died in prison between 2001 and 2020 (1 in the control group). 17 prison suicides were recorded from the study population; all occurred in the psychosis group."

3. **In the introduction, it would be helpful to include the effect estimates from the meta-analysis of suicide prevention programs in correctional facilities.**

Author's Response:
We have rewritten this sentence in the revised manuscript as follows:

Page 3:
"A review of 24 studies (1980–2022) found that suicide prevention programs in correctional facilities significantly reduced suicide deaths (RR = 0.35, 95% CI: 0.23-0.55), self-harm (Hedges' g = -0.54), and suicidal ideation (Hedges' g = -0.39), indicating their overall effectiveness despite variation in study designs and quality [8]."

4. **In the Methods, the matching with sex and age was done for the 143,506 cases? What was the age range for each match?**

Author's Response:
Yes, the matching with sex and age was done for the 143,506 individuals with psychosis. From the psychosis cohort and their matches, individuals with an incarceration record were selected

as cases and controls for the current study. We have explained this in the revised manuscript as mentioned in response **no. 1 (page 4)** above.

Also, note that birth year was used as the basis for matching from the MLK.

5. **Secondary outcomes should also be stated in the Methods.**

Author's Response:
Secondary outcomes have been included in the revised manuscript under methods section as follows:

> **Page 4:**
> "**Secondary outcome**
> The secondary outcome measures of this study was the application of alerts by Corrective Services in relation to self-harm incidents, and suicides in prison in the study population."

6. **The rationale for dichotomizing SEIFA should be provided (with at least a reference).**

Author's Response:
We agree and have included the rationale behind dichotomizing SEIFA also changed the wordings with a reference in the revised manuscript under covariates as follows:

> **Page 6:**
> "SEIFA was derived from both the APDC and EDDC. Socioeconomic status was assigned based on the Index of Relative Socio-economic Disadvantage (IRSD), which ranks areas according to relative disadvantage using Statistical Local Areas (SLAs) [12]. The lowest rank indicates the most disadvantaged areas, and the highest rank the most advantaged. We categorized areas into disadvantaged (lower 5 deciles 1–5) and advantaged (upper 5 deciles 6–10). We dichotomized SEIFA at the median (50-50 split) to create balanced groups for clearer comparison and improved statistical stability between relatively lower and higher socioeconomic disadvantage."

The reference for this is as follows **(page 16 in the revised manuscript):**

> 12. Australian Bureau of Statistics. (2021). Socio-Economic Indexes for Areas (SEIFA), Australia. ABS. https://www.abs.gov.au/statistics/people/people-and-communities/socio-economic-indexes-areas-seifa-australia/latest-release

7. **It is unclear if all tested predictors have been included in Tables 1 and 2; in addition, all predictors should be included in the Methods.**

Author's Response:

We confirm that all tested predictors were included in Tables 2 & 3 (note, table numbers have been changed) in the revised manuscript, and we have included a paragraph named Covariates in the revised manuscript under methods as follows:

> **Page 6:**
> **Covariates**
> We ascertained sex using the most commonly recorded value within and across the APDC, EDDC, OIMS, RBDM, and COD-URF datasets. Aboriginal status was determined based on any record indicating Aboriginality (Aboriginal or Torres Strait Islander status) in the service contact data, using the most frequent value within and across APDC, EDDC, OIMS, RBDM, and COD-URF. Age at first prison episode was calculated using date of birth and the start date of the first prison episode based on APDC, EDDC, and OIMS data and grouped into brackets: <25, 25–34, 35–44, and ≥45 years. Marital status (married or single) was sourced from the APDC, with "married" including de facto relationships, and "single" including those widowed, divorced, or permanently separated. SEIFA was derived from both the APDC and EDDC. Socioeconomic status was assigned based on the Index of Relative Socio-economic Disadvantage (IRSD), which ranks areas according to relative disadvantage using Statistical Local Areas (SLAs) [12]. The lowest rank indicates the most disadvantaged areas, and the highest rank the most advantaged. We categorized areas into disadvantaged (lower 5 deciles 1–5) and advantaged (upper 5 deciles 6–10). We dichotomized SEIFA at the median (50-50 split) to create balanced groups for clearer comparison and improved statistical stability between relatively lower and higher socioeconomic disadvantage.

8. Gender-stratified analyses should have been mentioned in the analysis methods.

Author's Response:
We have mentioned about the gender-stratified analysis in the revised manuscript as follows:

> **Page 7:**
> "Both combined and gender-stratified analyses were conducted for descriptive and logistic regression analysis."

9. Analysis methods for alerts need to be included.
- Sensitivity analyses on only participants with complete data should be carried out.

Author's Response:
We would like to mention that the analysis we did for the alerts was based on only those participants with complete prison episode data i.e., participants who completed a discrete prison episode (Figure 1; attached). Please see **page 10**:

"Alerts in completed prison episodes (2001-2020) in prisoners with a prior diagnosis of psychosis."

Additionally, we have mentioned about this in the statistical analysis section of the revised manuscript as follows:

Page 7:
"Prison alerts, self-harm and suicide were also reported only on study cohorts with completed prison episodes."

10. In the Results, the effect estimates and p-values for comparisons made regarding alerts and the most recent incarcerations need to be included.

Author's Response:
Thank you for this suggestion. We have run Chi-square tests and included p-values for comparisons related to alerts and the most recent incarcerations in the Results section of the revised manuscript. Please see section "Alerts in completed prison episodes (2001-2020) in prisoners with a prior diagnosis of psychosis" & "Self-harm and suicide during the most recent incarceration" on **page 10 & 12** in the revised manuscript. These additions provide greater clarity and support for the reported findings.

11. In the Discussion, the authors should clarify the impact of diagnosis made in private clinics, general practice, vs public services on the findings and how this impact was "likely small".

Author's Response:
We have mentioned about this in the revised manuscript in the limitations section and mentioned the reason for this factor having a small impact on the results as follows:

Page 14:
"This study did not include those diagnosed with psychosis exclusively in private clinics or by general practitioners, though most of those with psychosis are treated by public services at some point, so the impact of this on our findings is likely to be small."

12. The authors should also mention the lack of information to differentiate between non-suicidal self-injuries and suicide attempts with intent as a limitation.

Author's Response:
We have mentioned about this in the limitations in the revised manuscript as follows:

Page 14:
"This study was unable to distinguish between non-suicidal self-injury and actual suicide attempts with intent, which is another limitation of the available data."

13. **The authors could also elaborate on the comorbidities (limitations) in terms of psychiatric / medical conditions and consideration of these conditions in cases and controls.**

Author's Response:
Thank you for this comment. In our study, cases were defined as individuals with a history of a diagnosis of psychosis, while controls had no recorded history (i.e., diagnosis) of psychosis. We did not have data on other psychiatric or medical conditions for either group. As such, we were unable to account for additional comorbidities. We have now clarified this in the limitations section of the revised manuscript as follows:

Page 2:

- Data on other psychiatric or medical comorbidities were unavailable, limiting adjustment for these factors.

Page 14:
This study also lacked data on other psychiatric or medical comorbidities beyond psychosis, which limits our ability to account for their potential influence on the findings.

Reviewer 2: Prof. Zoe Kelson, University of Exeter

Comments to the Author:
The aim of this retrospective case-control data-linkage study was to examine self-harm behaviour in New South Wales (NSW) prisons in Australia among inmates diagnosed with psychosis from 2001 to 2020.

1. **Can the authors please further comment on whether they consider the included cohort to be representative?**

"143,506 cases were identified as individuals in NSW, Australia, diagnosed with psychosis on admission to hospital between July 2001 and June 2020 or an emergency department between June 2005 and June 2020. For this study, we selected 4,900 individuals from cases s who had a custodial episode in NSW prisons during July 2001 and June 2020."

Author's Response:
Thank you for the comment. In our study, the cases and controls represent the entire population meeting the inclusion criteria (i.e., all individuals with a record of an incarceration record from the with or without psychosis cohort), rather than a sample. Therefore, the issue of representativeness does not apply in this context**.** We have written this more clearly in the methods section under study population as mentioned earlier.

2. **Can the authors please specify the matching algorithm or process they followed here?**

"Two age- and sex-matched controls without any record of psychosis diagnosis were randomly selected for each case from the CheReL's (Centre for Health Record Linkage) control sampling frame"

Author's Response:

We have made changes in the control selection section where we mention the matching algorithm in the revised manuscript as follows:

> **Page 4:**
> **"***Controls:* Two age- and sex- matched individuals without any record of a psychosis diagnosis were randomly selected for each individual diagnosed with psychosis from the Centre for Health Record Linkage (CheReL's) control sampling frame using the random number generated by SAS [11]. Controls were matched to cases on year of birth and sex, and where the control's death date was either unknown (NULL), or greater than the case index date. The datasets included in the creation of this group without psychosis diagnosis were the NSW APDC, NSW EDDC, NSW Registry of Births, Deaths and Marriages (RBDM), NSW Mental Health Ambulatory Data Collection, NSW Perinatal Data Collection, NSW Cancer Registry, NSW Pap Test Registry, and NSW Notifiable Conditions Information Management System. CHeReL provided an additional 12,565 unlinked individual's data resulting in a total of 270,848 individuals as without any diagnosis of psychosis. Of these, 2,713 individuals had a custodial episode in NSW prisons during the same time period and considered as controls in this current study."

3. **Can analyses please also be stratified by control type (i.e. matched and unlinked)?**

"CHeReL also provided an additional 12,565 unlinked controls resulting in a total of 270,848 individuals as controls without any diagnosis of psychosis."
and
"For this study, we selected 2,713 controls who had a custodial episode in NSW prisons during the same time period. "

Author's Response:
Thank you for the suggestion. We believe that a stratified analysis by control type would offer limited additional insight, as we only have sociodemographic data available for the unlinked controls, with no further data on prison self-harm or alerts. In fact, the study population we got for this study (Cases: 14,900 individuals and controls: 2,713 individuals) from the larger cohort (143,506 individuals with psychosis and 270,848 individuals without any diagnosis of psychosis) does not include the unlinked controls as we only included those who had been incarcerated.

4. **Can the authors please confirm whether all incidents of self-harm and suicide have been captured in this data?**

"The primary outcome of this study was at least one self-harm incident in prison as recorded in the NSW Offender Integrated Management System (OIMS). We also examined prison alerts for self-harm incidents including suicide in prison."

Author's Response:
We confirm that all incidents of self-harm and suicide have been captured in this study. Please note that suicide records were extracted from the NSW Registry of Births, Deaths, and Marriages (RBDM) and Cause of Death Unit Record File (COD URF) and alerts and self-harm from the NSW Offender Integrated Management System (OIMS). We mention that for alerts, this study focused only on mental health and self-harm related alerts in the definition section as follows:

> **Page 7:**
> "This study focused on mental health related alerts and those concerning self-harm."

5. **Can the authors please clarify if (and how) distributional assumptions were checked prior to the application of non-parametric statistical descriptors?**

"Continuous variables were described using median and interquartile range (IQR)."

Author's Response:
Thank you for the question. We chose non-parametric statistical descriptors overall because most of our variables were categorical. For the continuous variable (age), we visually checked the distribution using histograms and Q-Q plots, which showed skewness. Given this, we used non-parametric summaries (like medians and interquartile ranges) to better represent the data. For the logistic regression analysis, normality of the variables is not required. Logistic regression is suitable for binary outcomes like self-harm (yes/no), and it models the relationship between the outcome and predictor variables through the log-odds. We used a forward stepwise approach to include statistically significant covariates in the model.

Note that due to word limits, we could not include all of these details in the manuscript, but we are happy to clarify them here.

6. **Appropriate modelling methods have been applied by the authors. Can the authors please specify (i.e. list) the covariates included in the model in the model in the methods section?**

"Multivariate regression analysis was used to examine predictors of self-harm in prison." [Abstract]
and
"Multivariable logistic regression was used to examine predictors of self-harm incidents in prison. Statistically significant covariates (p<0.05) were included in the model using a forward stepwise approach."

Author's Response:
We confirm that all covariates included in the model are described in the methods section. Please see **comment 7 above (page 6-7 in this document**) and **page 6** in the revised manuscript.

7. **Can the authors please comment on whether missing/unknown values can be assumed to be missing at random?**

"Unknown/missing values were combined with the most common category."

Author's Response:
We confirm that missing/unknown values can be assumed to be missing at random. Considering the moderate number of missing values in the variable SEIFA we have decided to keep the "missing/unknown" category in the logistic regression (Table 2 & Table 3) and mentioned this in the statistical analysis section of the revised manuscript as follows:

> **Page 7:**
> "We created a separate category for missing or unknown values."

8. **Can the authors please conduct sensitivity analyses on the approach taken for handling missing data (e.g. undertaking a complete case analysis and performing multiple imputation)?**

Author's Response:
Thank you for your suggestion. As mentioned above, we addressed missing data by creating a separate "missing/unknown" category for categorical variables with incomplete data (e.g., marital status, Aboriginal status, and SEIFA) in the revised manuscript. This approach allowed us to retain the full sample size and account for potential informative missingness, particularly given the moderate proportion of missingness in marital status (16%) and minimal missingness in the other variables. We acknowledge that alternative methods such as complete case analysis or multiple imputation could be considered in future research to assess the robustness of the findings.

9. **Thanks for providing a copy of the STROBE checklist in the supplementary material.**

"STROBE case-control reporting guidelines were used throughout the manuscript"

Author's Response:
We thank the reviewer for this. As per the Editor's suggestion we have made few changes in the checklist document. It has been attached with the manuscript.

10. Can the authors please confirm whether all significantly different variables between groups have been adjusted for in the models?

"Overall, 14,900 cases with psychosis and 2,713 matched controls were incarcerated at least once between July 2001 and June 2020 (Table 1)."
and
"24.5% of cases were of Aboriginal heritage compared to 20.2% of controls. A higher proportion of controls reported having married or de facto relationship status (22.0% vs. 11.5%) and being from a disadvantaged area was less common in controls compared to cases (62.8% vs. 69.5%)."

"a greater proportion of women in the control group were aged between 35-44 years compared to cases (34.6% vs. 22.4%). Among both cases and controls, a greater proportion of women were Aboriginal compared to men (35.8% vs. 22.2% in cases; 27.0% vs. 19.6% in controls); and among cases, a higher proportion of women were married/in a de facto relationship compared to men (14.0% vs. 11.0%)"

Author's Response:
We confirm that all significantly different variables between groups (shown in Table 2 in the revised manuscript) have been adjusted for in the models (Table 3) in the revised manuscript. We have included the following in the revised manuscript as well:

> **Page 10:**
> "Table 3 presents the factors associated with an increased risk of self-harm, showing adjusted odds ratios (aORs) and 95% confidence intervals. All estimates are mutually adjusted for the variables listed in the table."

Please see our response in **Point 13 below for the revised Table.**

11. Can the fact that 'all of the suicides occurred in the psychosis group' please be clarified in the Abstract?

"35 individuals with psychosis died in prison between 2000 and 2020; 17 from suicide" [Abstract]
and

"A total 17 suicides were reported inside prison among the study population and all of the suicides occurred in the psychosis group"

Author's Response:
This has been clarified in the Abstract in the revised manuscript as follows:

> **Page 2:**
> "35.6% of those released from prison with a prior psychosis had at least one alert applied during incarceration compared to 10.1% of prisoner without a diagnosis of psychosis. Overall, 35 individuals with psychosis died in prison between 2001 and 2020 (1 in the control group). 17 prison suicides were recorded from the study population; all occurred in the psychosis group."

12. Can the number of individuals without psychosis who died in prison between 2000 and 2020 please also be reported (as proportions)?

Author's Response:

We have mentioned this in the abstract **(see response above)** and also in detail under the "Self-harm and suicide during the most recent incarceration" section in the revised manuscript as follows:

> **Page 12:**
> "Overall, from this study cohort a total of 1209 cases and 132 controls died up until June 2019. Among these, 35 individuals with psychosis (0.23% of cases) died whilst in prison compared to 1 control (0.04%) indicating almost a 6-fold higher rate of in-prison deaths among cases compared with controls, although this difference was not statistically significant (p = 0.25)."

13. Can the table heading please specify that Marital status and Socio-Economic Indices for Areas have also been adjusted for in the models?

Table 2: "adjusted by psychosis type, age, sex, and Aboriginal background"

Author's Response:
We agree and have redone this analysis **(Table 3 in the revised manuscript)** with all covariates adjusted in the revised manuscript. We would like to mention that the results are almost similar with previous results. Please see **page 11** in the revised manuscript as follows:

Table 3. Adjusted Odds ratios (aOR) for self-harm in prison- July 2001-June 2020

	Overall (n = 17,613)			Men (n = 14,869; 84.4%)		Women (n = 2,744; 15.6%)	
	Self-harm n (%)	aOR (95% CI)	p-value	aOR (95% CI)	p-value	aOR (95% CI)	p-value
**Psychosis**							
No	98 (3.6)	1		1		1	
Schizophrenia and related psychoses	1,921 (16.3)	4.84 (3.93-5.98)	<0.001	4.77 (3.83-5.95)	<0.001	5.71 (2.83-11.10)	<0.001
Affective psychosis	52 (11.5)	3.72 (2.60-5.32)	<0.001	4.17 (2.83-6.15)	<0.001	2.62 (1.00-6.91)	0.051
Substance-related psychosis	257 (9.6)	2.51 (1.97-3.19)	<0.001	2.48 (1.91-3.21)	<0.001	2.82 (1.36-5.86)	0.006
**Gender**							
Female	487 (17.7)	1		-		-	
Male	1,841 (12.4)	0.72 (0.64-0.81)	<0.001	-	-	-	-
**Indigenous status**							
No	1,514 (11.3)	1		1		1	
Yes	813 (19.4)	1.58 (1.43-1.75)	<0.001	1.70 (1.52-1.90)	<0.001	1.23 (1.00-1.51)	0.055
Missing/unknown	1 (4.8)	0.39 (0.05-2.95)	0.359	1	-	1.92 (0.16-23.27)	0.609
**Age groups at first prison episode**							
<25 years	1,169 (19.7)	1		1		1	
25-34 years	787 (12.0)	0.56 (0.51-0.63)	<0.001	0.60 (0.54-0.67)	<0.001	0.44 (0.35-0.55)	<0.001
35-44 years	290 (8.0)	0.37 (0.32-0.43)	<0.001	0.38 (0.32-0.44)	<0.001	0.34 (0.25-0.46)	<0.001
45+ years	82 (5.4)	0.25 (0.20-0.32)	<0.001	0.28 (0.22-0.37)	<0.001	0.14 (0.07-0.25)	<0.001
**Marital status**							
Married	290 (12.5)	1		1		1	
Single	1,728 (13.9)	0.89 (0.77-1.01)	0.072	0.86 (0.74-1.01)	0.069	0.95 (0.70-1.27)	0.707
Missing/unknown	310 (10.9)	0.73 (0.62-0.88)	0.001	0.69 (0.56-0.84)	<0.001	0.94 (0.64-1.36)	0.732
**SEIFA**							
Advantaged	660 (12.3)	1		1		1	
Disadvantaged	1,507 (13.4)	1.00 (0.91-1.11)	0.949	0.94 (0.84-1.05)	0.286	1.29 (1.02-1.63)	0.031
Missing/unknown	161 (16.2)	1.27 (1.05-1.54)	0.015	1.25 (1.01-1.54)	0.037	1.31 (0.80-2.12)	0.280

Note that, following updates to the table, some effect estimates, and p-values have changed slightly (please see section self-harm in prison on **page 10** in the revised manuscript). However, these changes did not alter the overall findings or interpretations of the study.

14. A discussion on the study limitations has been provided.

"This study excluded those diagnosed with psychosis exclusively in private clinics or by general practitioners, though most are treated by public services at some point, so the impact is likely small. It only includes NSW records, so those diagnosed with psychosis or convicted of a crime interstate or overseas would have been missed. Alerts and self-harm incidents recorded by prison staff may be prone to human error, bias, and reporting reluctance can affect accuracy. The study also lacks data on ethnic groups, employment/education history, and other comorbidities."

Author's Response:
In response to **Editors comment (no 3),** we have also mentioned some of the limitations just after the abstract and included in the "Strengths and limitations of this study" in the revised manuscript mentioned above. Also, we have changed the wordings in the limitations section in the revised manuscript as follows:

> **Page 14:**
> "**Limitations**
> This study did not include those diagnosed with psychosis exclusively in private clinics or by general practitioners, though most of those with psychosis are treated by public services at some point, so the impact of this on our findings is likely to be small. Our study only included records for NSW, so those diagnosed with psychosis or convicted of a crime in another state or overseas would not have been included. Alerts and self-harm incidents recorded by prison staff may be subject to reporting bias, underreporting, and human error, which could affect the accuracy and completeness of the data. This study also lacked data on other psychiatric or medical comorbidities beyond psychosis, which limits our ability to account for their potential influence on the findings. This study was unable to distinguish between non-suicidal self-injury and actual suicide attempts with intent, which is another limitation of the available data."

VERSION 2 - REVIEW

**Reviewer**	**2**
**Name**	**Kelson, Zoe**
**Affiliation**	**University of Exeter, Mathematics**

Date **17-Jul-2025**

COI

Thanks to the authors for their responses and revisions.

The following reviewer requests are raised:

Author response:

"We believe that a stratified analysis by control type would offer limited additional insight, as we only have sociodemographic data available for the unlinked controls, with no further data on prison self-harm or alerts. In fact, the study population we got for this study (Cases: 14,900 individuals and controls: 2,713 individuals) from the larger cohort (143,506 individuals with psychosis and 270,848 individuals without any diagnosis of psychosis) does not include the unlinked controls as we only included those who had been incarcerated."

Reviewer request:

Can this please be clarified in the article?

Author response:

"For the continuous variable (age), we visually checked the distribution using histograms and Q-Q plots, which showed skewness."

Reviewer request:

Can this please be incorporated into the article?

Author response:

"We confirm that missing/unknown values can be assumed to be missing at random"

Reviewer request:

Can this please be noted in the article, along with a justification for this assumption?

VERSION 2 - AUTHOR RESPONSE

Reviewer: 2
Prof. Zoe Kelson, University of Exeter
Comments to the Author:
Thanks to the authors for their responses and revisions.
The following reviewer requests are raised:

1. Author response: "We believe that a stratified analysis by control type would offer limited additional insight, as we only have sociodemographic data available for the unlinked controls, with no further data on prison self-harm or alerts. In fact, the study population we got for this study (Cases: 14,900 individuals and controls: 2,713 individuals) from the larger cohort (143,506 individuals with psychosis and 270,848 individuals without any diagnosis of psychosis) does not include the unlinked controls as we only included those who had been

incarcerated."
Reviewer request:

Can this please be clarified in the article?

Author’s response:
We have clarified this in the revised manuscript as follows:

Page 4:
“Of these, 2,713 individuals had a custodial episode in NSW prisons during the same time period and considered as controls in this current study which excludes the unlinked controls, including only incarcerated individuals.”

2. Author response:
"For the continuous variable (age), we visually checked the distribution using histograms and Q-Q plots, which showed skewness."
Reviewer request:
Can this please be incorporated into the article?

Author’s response:
We have incorporated this into the article as follows:
Page 7:
“Continuous variables were described using median (IQR) due to skewed distributions observed in histograms and Q-Q plots.”

3. Author response:
"We confirm that missing/unknown values can be assumed to be missing at random"
Reviewer request:
Can this please be noted in the article, along with a justification for this assumption?

Author’s response:
We have mentioned this in the revised manuscript with a justification for this assumption as follows:

Page 7-8:
“Missing data were assumed to be missing at random, as missingness was associated with observed sociodemographic variables rather than unobserved outcomes. We created a separate category for missing or unknown values.”

4. Reviewer: 2
If you have selected ‘Yes’ above, please provide details of any competing interests.: Not applicable

Author’s response:
We are unable to understand the reviewer’s comment or the section to which it refers. However, we have mentioned the competing interests in the manuscript as follows:

Page 15:
“Competing Interests Statement
TB has received research funding from Corrective Services NSW. LG is employed by Corrective Services NSW. NC, JH, ES, AK, KD, AA, AE, DG, and SA have no competing interests to declare.”
